# Gender specific excess mortality in Italy during the COVID-19 pandemic accounting for age

**DOI:** 10.1007/s10654-021-00717-9

**Published:** 2021-01-25

**Authors:** Emilio A. L. Gianicolo, Antonello Russo, Britta Büchler, Katherine Taylor, Andreas Stang, Maria Blettner

**Affiliations:** 1 Institute for Medical Biostatistics, Epidemiology and Informatics (IMBEI), University Medical Center of the Johannes Gutenberg University of Mainz, Mainz, Germany; 2 Institute of Clinical Physiology of the Italian National Research Council (IFC-CNR), Lecce, Italy; 3 Institute of Atmospheric Sciences and Climate of the Italian National Research Council (CNR-ISAC), Lecce, Italy; 4 Institute of Medical Informatics, Biometry and Epidemiology, University Hospital Essen, Essen, Germany; 5 Department of Epidemiology, School of Public Health, Boston University, Boston, USA

**Keywords:** COVID-19, Mortality, Italy, age standardisation, Sex differences

## Abstract

Since the beginning of the COVID-19 pandemic, data have been accumulated to examine excess mortality in the first half of 2020. Mortality in the preceding year or years is used to calculate the expected number of deaths, which is then compared with the actual number of deaths in 2020. We calculated weekly age- and sex-specific mortality rates for 93.1% of the Italian municipalities for the years 2015–2019 and for the first 26 weeks in 2020. We assumed the mortality experience during 2015–2019 as the reference period to calculate standardised mortality ratios. Furthermore, in order to compare the mortality experience of males and females, we calculated sex- and age- specific weekly direct standardised mortality rates and differences between the observed and expected number of deaths. We observed considerable changes in the demographics in the Italian population between the years 2015 and 2020, particularly among people 60 years and older and among males. The population is aging and the proportion of elderly males has increased, which was not reflected adequately in previous estimates of excess mortality. Standardized excess mortality results show that in Italy between the 8th and 26th weeks in 2020, there were 33,035 excess deaths, which is only 643 fewer deaths than the official COVID-19 death toll for this time period. A comparative increase in the mortality rates was observed in March among both sexes, but particularly for males. Comparisons with recently published data show considerably higher excess deaths, but these data were either not covering the complete country or did not account for age and sex. Neglecting the demographic changes in a region, even over a short time span, can result in biased estimates.

**Electronic supplementary material:**

The online version of this article (10.1007/s10654-021-00717-9) contains supplementary material, which is available to authorized users.

## Introduction

COVID-19, the disease associated with Severe Acute Respiratory Coronavirus 2 (SARS-CoV-2), was first reported in Wuhan (Hubei Province, China), and as of 9 October 2020, 36.4 million confirmed cases and over 1 million deaths have been reported worldwide [1]. Health care facilities, in particular intensive care units, in some countries have been overwhelmed with COVID-19 patients. Due to the lack of effective treatment strategies or a vaccine, broad public health control measures have been recommended and implemented in most countries in order to reduce the transmission of SARS-CoV-2.

Countries have shown varying patterns in the spread of COVID-19, and Italy—in particular its northern region of Lombardy—was among the most affected European countries. However, comparing incidence and mortality between countries is difficult due to high heterogeneity of testing strategies, testing potential of laboratories, and demographic differences. This short article investigates the influence of the last point,

Official COVID-19 death registries only count deaths directly caused by COVID-19 (direct mortality). However, indirect mortality, referring to deaths not directly caused by the COVID-19 disease but through circumstances caused by the COVID-19 pandemic (for example caused by overburdened health care systems) should also be considered. Thus, an excess of the overall mortality within a population can be an indicator of the impact of COVID-19. In Italy, data on excess mortality have been reported but only on a restricted number of cities or regions [2, 3] or analyses have not accounted for age and sex standardisation [4]. The latter point is particularly important both from an epidemiological and a public health point of view. While comparing populations over time, the evolving age structure must be considered. For example, as shown by Stang et al., in Germany the proportion of the oldest people (e.g. in the age-group 80 years and older) has dramatically increased from 2016 to 2019 (+17.1%) [5]. In this age group COVID-19 was particularly lethal [6]. Therefore, ignoring changes of the age structure over time may result in a biased estimation of excess mortality. Sex-specific data is also paramount when dealing with COVID-19-related mortality, since different patterns of COVID-19 mortality have been shown among males and females [7]. Daily sex- and age-specific all-cause mortality numbers in Italy from January to June 2020 are available from the Italian National Institute of Statistics [8]. The aim of our study was to calculate sex- and age-specific weekly standardised mortality ratios and rates and estimate the excess of mortality during the pandemic using as references the period of 2015–2019 in Italy and the European standard population respectively. Furthermore, we compared the estimated excess of mortality with the officially registered COVID-19 mortality.

## Materials and methods

### Mortality and population data

On August 10, 2020, the Italian National Institute of Statistics (ISTAT) published daily sex- and age-specific mortality data up to 30 June (26th calendar week). ISTAT obtained these figures by integrating population (Anagrafe Nazionale della Popolazione Residente) and national tax registries (Anagrafe tributaria) [9]. For 537 municipalities this integration was evaluated by ISTAT as unreliable. However, figures were available for 7357 of 7904 municipalities (93.1%).

To determine the number of officially recorded COVID-19 deaths in Italy, we used data from the Italian National Institute of Health, which recorded the first death on 21 February 2020 (8th calendar week). Due to this, our study period covers the time span: from the 8th to the 26th calendar week of 2020 (February 21–June 30). COVID-19 deaths were confirmed by the Italian National Institute of Health, which followed the criteria established by the WHO [9].

Weekly population figures of the Italian population are not available. Therefore, we used population numbers published by ISTAT as of 1 January of each year to calculate sex- and age-specific weekly mortality rates. We did not consider populations living in those municipalities (n = 537) for which ISTAT did not provide mortality data.

### Standardised mortality ratios (SMR)

Weekly average mortality ratios for the 8th to the 26th weeks for the period 2015–2019 were calculated by dividing the weekly sex- and age-specific (0–29, 30–49, 50–59, 60–69, 70–79, 80+ years) number of deaths in each of the years 2015–2019 by the sex and age-specific average populations for the relevant year. Thereafter, we averaged the sex- and age-specific rates for the overall period of 2015–2019 and multiplied these rates by the age- and sex-specific population in Italy as of 1 January 2020, to calculate the expected weekly number of deaths in 2020 for the calendar weeks 8–26. SMRs were calculated by dividing observed and expected number of deaths. We multiplied these values by 100. 95% confidence intervals (95% CI) were calculated based on the Byar’s approximation [10].

### Differences between COVID-19 deaths and the average number of deaths for the period 2015–2019

Weekly excess mortality for the weeks 8–26 in 2020 was calculated as the difference between the observed and expected number of deaths.

For the same period, we calculated differences between the sex- and age-specific estimated number of excess deaths and the numbers of COVD-19 deaths as officially reported by the Italian National Institute of Health. Finally, we calculated the difference between the average number of deaths from 2015 to 2019 and the expected number of deaths.

### Direct standardised mortality rates and sex ratios of age-standardised mortality rates

In order to compare the mortality experience of males and females, for 2020 and for the period 2015–2019 we calculated sex- and age-specific weekly direct standardised mortality rates and related 95% confidence intervals assuming the standard European population as the Ref. [11]. Furthermore, to quantify sex differences in age-standardised mortality rates, we calculated sex ratios of age-standardised mortality rates with women in the denominator.

## Results

### Demographic changes in Italy over the years 2015–2020

Using demographic data as of 1 January of each year, in Italy the group aged 80+ years increased from 3,977,449 in 2015 to 4,442,048 in 2020 (+11.7%), and the population over 60 years old increased from 16,849,329 to 17,874,053 in the same time period (6.1%) (Table 1). Sex-specific figures also show a considerable change, in particular with the proportion of males over 80 years old, which increased by 17.5% from 2015 to 2020 compared to 8.4% for females over 80 years old (Table 1).

**Table 1:** Population of Italy by age and sex from 2015 to 2020 as of 1 January of each year

Age groups (years)	2015	2016	2017	2018	2019	2020	Percentage change^a^
*Males*							
0–29	9,033,691	8,968,422	8,917,180	8,881,148	8,809,898	8,716,257	− 3.5
30–49	8,812,266	8,641,810	8,472,187	8,306,681	8,152,940	7,996,011	− 9.3
50–59	4,229,923	4,327,588	4,424,956	4,506,789	4,578,610	4,656,253	10.1
60–69	3,447,664	3,512,422	3,507,570	3,511,156	3,511,037	3,554,434	3.1
70–79	2,562,600	2,550,154	2,624,947	2,680,724	2,727,000	2,753,864	7.5
80+	1,415,446	1,455,925	1,498,901	1,541,109	1,605,281	1,663,746	17.5
Total	29,501,590	29,456,321	29,445,741	29,427,607	29,384,766	29,340,565	− 0.5
*Females*							
0–29	8,578,416	8,491,875	8,410,523	8,332,551	8,250,728	8,160,630	− 4.9
30–49	8,857,074	8,688,700	8,507,209	8,328,661	8,163,932	7,996,508	− 9.7
50–59	4,434,913	4,531,162	4,624,040	4,705,957	4,773,621	4,844,927	9.2
60–69	3,743,962	3,818,736	3,816,067	3,823,808	3,826,173	3,870,741	3.4
70–79	3,117,654	3,085,625	3,152,112	3,199,498	3,235,533	3,252,966	4.3
80+	2,562,003	2,593,132	2,633,753	2,665,891	2,724,793	2,778,302	8.4
Total	31,294,022	31,209,230	31,143,704	31,056,366	30,974,780	30,904,074	− 1.2
*Males and females*							
0–29	17,612,107	17,460,297	17,327,703	17,213,699	17,060,626	16,876,887	− 4.2
30–49	17,669,340	17,330,510	16,979,396	16,635,342	16,316,872	15,992,519	− 9.5
50–59	8,664,836	8,858,750	9,048,996	9,212,746	9,352,231	9,501,180	9.7
60–69	7,191,626	7,331,158	7,323,637	7,334,964	7,337,210	7,425,175	3.2
70–79	5,680,254	5,635,779	5,777,059	5,880,222	5,962,533	6,006,830	5.7
80+	3,977,449	4,049,057	4,132,654	4,207,000	4,330,074	4,442,048	11.7
Total	60,795,612	60,665,551	60,589,445	60,483,973	60,359,546	60,244,639	− 0.9

^**a**^Percentage change from 2015 to 2020

### Standardised mortality ratios (SMR)

**Fig. 1:**
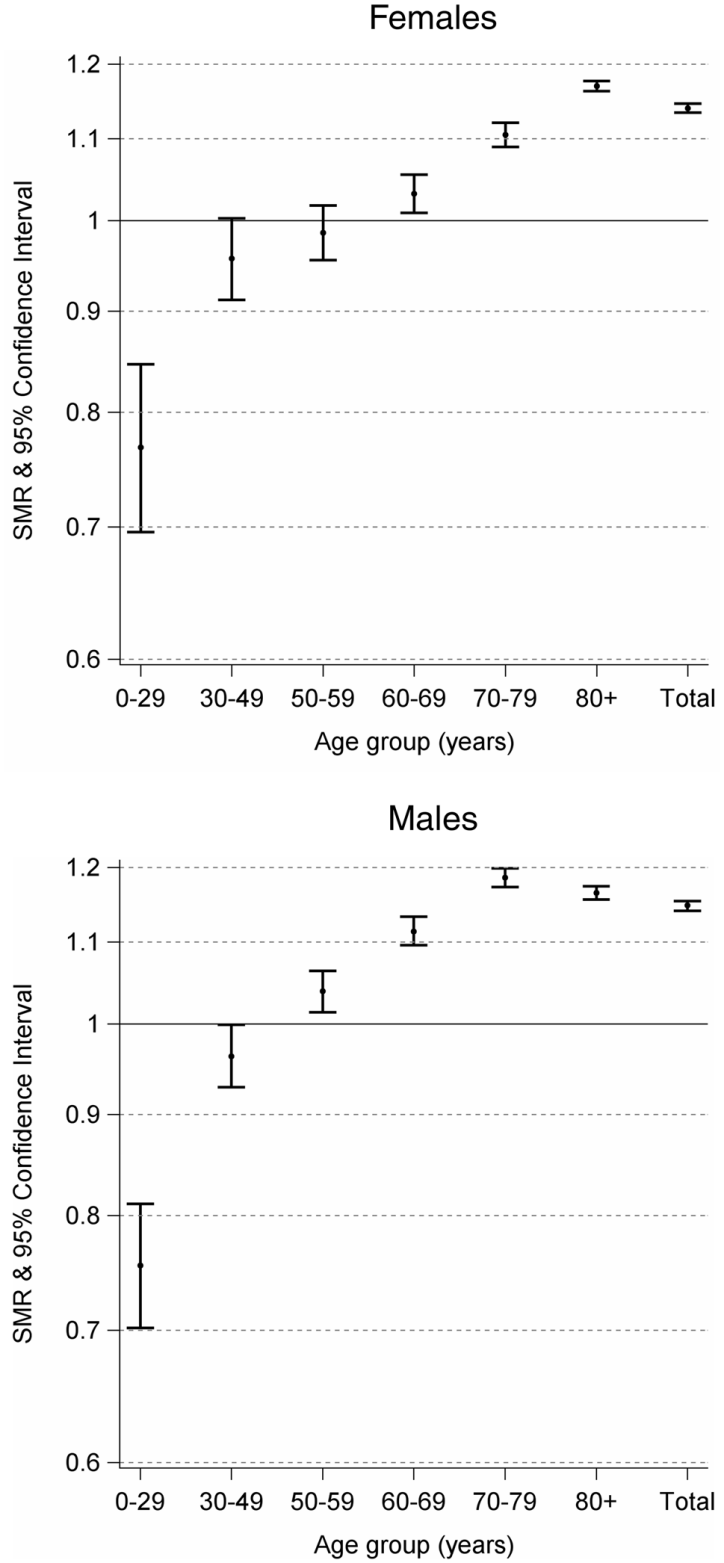
Standardised mortality ratios (SMR) on a logaritmic scale and 95% CI by age and sex in Italy for calendar weeks 8–26. Expected numbers are calculated with 2015–2019 as the reference period

In the reference period 2015–2019, the average number of deaths in the time span from the 8th to the 26th calendar week was 219,064 (Table 2). The expected number of deaths was 229,864, which amounts to an overall SMR of 114.4 (95%IC 113.9–114.8) (Fig. 1). SMRs lower than 100 were observed in the youngest age groups for both males and females (Fig. 1). We found a difference of 33,035 between observed (262,899) and expected number (229,864) of deaths between the 8th and the 26th calendar week. The oldest age groups (60+ years) drove this excess mortality (Table 2 and Fig. 1). In the same period, the Italian National Institute of Health registered 33,678 COVID-19 deaths (Table 2).

**Table 2:** Average number of deaths from 2015 to 2019, number of deaths in 2020, and expected number of deaths in 2020 by age and sex in Italy for calendar weeks 8–26

Age groups (years)	Average number of deaths 2015–2019 (A)	Number of deaths observed in 2020 (B)	Number of deaths expected in 2020 (C)	Number of officially registered as death COVID-19 (D)	Number of observed deaths in 2020—expected (B − C)	Difference between number of observed deaths and average number of deaths (B − A)	Number of observed deaths in 2020—expected—officially death COVID-19 (B − C − D)
*Male*							
0–29	1015	752	995	13	− 243	− 263	− 256
30–49	3201	2935	3047	255	− 112	− 266	− 367
50–59	6022	6542	6298	892	244	520	− 648
60–69	12,839	14,489	13,003	2597	1486	1650	− 1111
70–79	25,944	31,989	26,983	6194	5006	6045	− 1188
80+	55,825	70,930	60,888	9572	10,042	15,105	470
Total	104,845	127,637	111,215	19,523	16,422	22,792	− 3101
*Female*							
0–29	555	416	541	7	− 125	− 139	− 132
30–49	1872	1700	1777	105	− 77	− 172	− 182
50–59	3660	3758	3811	281	− 53	98	− 334
60–69	7455	7793	7555	810	238	338	− 572
70–79	17,943	20,344	18,405	2706	1939	2401	− 767
80+	82,734	101,251	86,561	10,246	14,690	18,517	4444
Total	114,219	135,262	118,650	14,155	16,612	21,043	2457
*Total*							
0–29	1570	1168	1537	20	− 369	− 402	− 389
30–49	5073	4635	4824	360	− 189	− 438	− 549
50–59	9681	10,300	10,109	1173	191	619	− 982
60–69	20,294	22,282	20,558	3407	1724	1988	− 1683
70–79	43,887	52,333	45,388	8900	6945	8446	− 1955
80+	138,559	172,181	147,449	19,818	24,732	33,622	4914
Total	219,064	262,899	229,864	33,678	33,035	43,835	− 643

### Direct standardised mortality rates and sex ratios of age-standardised mortality rates

**Fig. 2:**
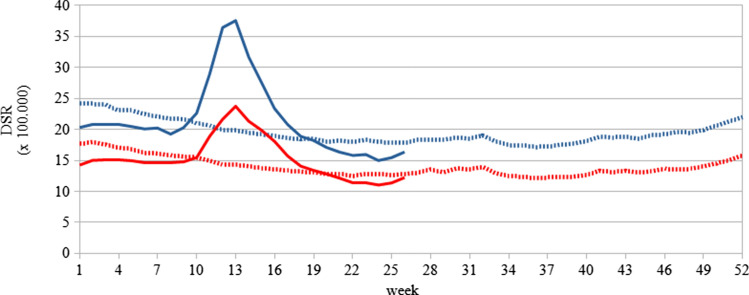
Direct standardised mortality rates (DSR) per 100,000 at the baseline (2015–2019 dotted line) and in 2020 (solid line) for males (blue lines) and females (red lines) Italy, calendar weeks from the 8th to the 26th

During the 9th–17th calendar weeks, both males and females showed increased direct standardised rates (Fig. 2). On average the standardised rate in the first 8 calendar weeks in the period 2015–2019 was equal to 16.9 and to 23.1 per 100,000 among females and males respectively (data not shown). In the same calendar week in 2020, the mortality rates were lower among males and females (females 14.8; males 20.3). Reduced rates were also observed during the fall and winter months in 2019 (Fig. 2). However, during the 9th–17th calendar weeks, mortality increased dramatically, peaking in the 13th week when mortality rates were equal to 23.7 and 37.5 among females and males respectively (Fig. 2).

During the reference period 2015–2019, standardised mortality rates were higher among males than among females (average sex ratio of age-standardised mortality rates in the weeks 1-26–1.39, range 1.34–1.44). However, the sex ratio increased markedly between the 10th and the 14th week, reaching its maximum in the 12th week (1.69).

## Discussion

In the time periods under study, we found a crude excess mortality of 43,835 when comparing the average number of deaths in the reference period 2015–2019 with the number observed in 2020. However, if the evolving demographic structure is taken into consideration, this difference becomes much smaller (33,035) with only 643 fewer deaths than the official number of COVID-19 deaths.

Alicandro et al. found 44,107 more deaths occurred between March and May 2020 (from week 10 to week 22) compared to the average number of deaths in these months between 2015 and 2019. This is 10,721 more deaths than officially recognized COVID-19 deaths in this time period. The authors partially attributed this difference to under-identification of COVID-19 deaths. However, Alicandro et al. used the average deaths in 2015-2019 without considering changes of the age- and sex-distribution over time. Using age standardisation, we estimated 7414 fewer deaths than Alicandro et al. in the same period (data not reported in tables).

### Limitations and strengths of the study

While interpreting the difference between the estimated excess mortality and the officially registered COVID-19 deaths, the lack of 100% mortality reporting should be considered. Since municipalities for which the Italian National Institute of Statistics did not provide daily all-cause mortality data account for 5.2% of the total mortality in the years 2015–2019, the difference between the excess of mortality and the number of deaths officially registered as COVID-19 might be underestimated.

Furthermore, the choice of a reference period is somewhat arbitrary in nature; a different reference may have produced different results. For instance, removing from the reference period a year which presented high mortality rates because of seasonal influenza would reduce the estimated expected number of deaths.

While a considerable portion of the excess mortality is likely a direct effect of the COVID-19, indirect effects are also important. During the country-wide lock-down in Italy, access to healthcare was limited, and residents had medical procedures cancelled or delayed. The psychological effects of lock-down and coping mechanisms such as increased drug and alcohol abuse may also have a role in the excess mortality [12]. On the other hand, with the lock-down, fewer deaths may have occurred for other reasons, for example due to fewer car accidents and work-related accidents. This is a possible explanation for the reduction in deaths in 2020 among the younger age groups in Italy, particular among men but also for women. One should also consider changes in mortality patterns before the onset of COVID-19 in the Italian population. For example, a warmer 2019 winter and a mild influenza season preceding January 2020 means the number of people particularly vulnerable to COVID-19 was higher than it might have been had these aspects been different.

ISTAT could not provide general mortality data for 537 municipalities; however, there is no evidence that these municipalities are clustered in specific regions. Thus, we can assume that data available is representative of the whole country. Furthermore, only yearly population data was considered for the analysis. We could not use weekly population figures to estimates the weekly mortality rates in 2020 because these figures are not available in a timely manner.

## Conclusion

In conclusion, taking into account the sex and age structure of the population is essential when reporting on mortality data and comparing different time spans. In future, cause-specific analyses might shed light on different patterns in mortality across the Italian population during the pandemic.

## Electronic supplementary material

Below is the link to the electronic supplementary material.

Supplementary material 1 (DOCX 16 kb)
