# Supplementary material for: Gender specific excess mortality in Italy during the COVID-19 pandemic accounting for age
Source: Eur J Epidemiol. 2021 Jan 25;36(2):213–8. doi: 10.1007/s10654-021-00717-9 (PMC7832414; doi:10.1007/s10654-021-00717-9)
Supplement: Supplementary file 1 — Supplementary material 1 (DOCX 16 kb) [file 10654_2021_717_MOESM1_ESM.docx]

**Supplementary material. Table S1. Sex ratios of age-standardised mortality rates and 95% confidence intervals (CI) in Italy. Females are the reference.**

| Week | Baseline 2015-2019 | | |  | 2020 | | |
| --- | --- | --- | --- | --- | --- | --- | --- |
|  | Sex ratios of age-standardised mortality rates and  95% CI | | |  | Sex ratios of age-standardised mortality rates  and 95% CI | | |
| 1 | 136.7 | (130.5- 143.2) | |  | 142.4 | (135.5 - 149.7) | |
| 2 | 134.3 | (128.2 -140.6) | |  | 138.3 | (131.7 - 145.3) | |
| 3 | 136.8 | (130.6 -143.3) | |  | 138.4 | (131.8 - 145.3) | |
| 4 | 135.1 | (128.9 -141.6) | |  | 137.9 | (131.3 - 144.8) | |
| 5 | 137.0 | (130.7 -143.7) | |  | 136.9 | (130.3 - 143.8) | |
| 6 | 138.3 | (131.8 -145.1) | |  | 137.2 | (130.5 - 144.2) | |
| 7 | 137.1 | (130.6 -143.9) | |  | 138.2 | (131.5 - 145.2) | |
| 8 | 137.5 | (130.9 -144.3) | |  | 132.0 | (125.6 - 138.8) | |
| 9 | 138.5 | (131.8 -145.4) | |  | 137.3 | (130.7 - 144.3) | |
| 10 | 136.0 | (129.4 -142.9) | |  | 146.4 | (139.6 - 153.5) | |
| 11 | 138.1 | (131.3 -145.2) | |  | 153.2 | (146.8 - 159.9) | |
| 12 | 138.9 | (131.9 -146.2) | |  | 168.5 | (162.1 - 175.1) | |
| 13 | 138.6 | (131.6 -145.9) | |  | 158.2 | (152.4 - 164.3) | |
| 14 | 138.6 | (131.6 -145.9) | |  | 148.6 | (142.8 - 154.7) | |
| 15 | 139.7 | (132.6 -147.2) | |  | 138.7 | (132.9 - 144.7) | |
| 16 | 139.8 | (132.6 -147.4) | |  | 129.3 | (123.6 - 135.2) | |
| 17 | 139.3 | (132.1 -146.9) | |  | 132.4 | (126.1 - 138.9) | |
| 18 | 139.6 | (132.4 -147.3) | |  | 134.3 | (127.6 - 141.3) | |
| 19 | 141.3 | (134.0 -149.1) | |  | 136.1 | (129.2 - 143.3) | |
| 20 | 140.5 | (133.1 -148.3) | |  | 133.5 | (126.6 - 140.9) | |
| 21 | 142.0 | (134.5 -149.8) | |  | 134.6 | (127.4 - 142.2) | |
| 22 | 144.1 | (136.5 -152.2) | |  | 138.3 | (130.7 - 146.3) | |
| 23 | 142.5 | (135.1 -150.4) | |  | 139.4 | (131.8 - 147.4) | |
| 24 | 139.7 | (132.4 -147.5) | |  | 135.8 | (128.2 - 143.8) | |
| 25 | 141.9 | (134.4 -149.8) | |  | 135.9 | (128.4 - 143.8) | |
| 26 | 139.2 | (131.9 -147.0) | |  | 133.6 | (126.5 - 141.1) | |
| **Min** | **134.3** |  |  |  | **129.3** |  |  |
| **Max** | **144.1** |  |  |  | **168.5** |  |  |
